# Tumor-Associated Glycans and Immune Surveillance 

**DOI:** 10.3390/vaccines1020174

**Published:** 2013-06-17

**Authors:** Behjatolah Monzavi-Karbassi, Anastas Pashov, Thomas Kieber-Emmons

**Affiliations:** 1 Winthrop P. Rockefeller Cancer Institute and Department of Pathology, University of Arkansas for Medical Sciences, Little Rock, AR 72205, USA; 2 Stephan Angeloff Institute of Microbiology, BAS, Sofia 1113, Bulgaria

**Keywords:** monoclonal antibodies, immunotherapy, cancer, mimics, vaccine, TACA, glycans, tumor, carbohydrate

## Abstract

Changes in cell surface glycosylation are a hallmark of the transition from normal to inflamed and neoplastic tissue. Tumor-associated carbohydrate antigens (TACAs) challenge our understanding of immune tolerance, while functioning as immune targets that bridge innate immune surveillance and adaptive antitumor immunity in clinical applications. T-cells, being a part of the adaptive immune response, are the most popular component of the immune system considered for targeting tumor cells. However, for TACAs, T-cells take a back seat to antibodies and natural killer cells as first-line innate defense mechanisms. Here, we briefly highlight the rationale associated with the relative importance of the immune surveillance machinery that might be applicable for developing therapeutics.

## 1. Introduction

A basic premise underlying immune modalities for cancer is that the immune system can mount a rejection strength response against neoplastically transformed cells [1]. Tumor targeting draws upon two immunological mediated paradigms. The first draws upon concepts of immune surveillance that bridges both innate and adaptive immunity. According to the immune surveillance hypothesis, tumor associated antigens are regarded as “non-self” by the immune system, and a major function of the immune system is to survey the body for the development of malignancy and to eliminate tumor cells as they arise [2]. Innate immunity relies on biochemical and cellular defense mechanisms often observed in the early phases of encounter with microbes. The cellular players include natural killer (NK) cells, dendritic cells (DCs), macrophages, monocytes, γδ T-cells and natural killer T (NKT)-cells. Adaptive immunity involves the expansion of T-cells and B-cells and their humoral and cellular mediators, cytokines and antibodies. In particular, antibodies and NK cells are early participants in the immune response and are particularly effective in eliminating blood-borne metastases [3]. In contrast, T-cells are the effector cells responsible for specific, long-lasting immunity.

The second draws upon concepts associated with tissue-specific destruction in the context of acute allograft (acute) rejection, flares of autoimmunity and response to acute infection. This second paradigm requires an understanding of the distinct difference between an anti-tumor immune response and outright tumor rejection. In this context, immune-mediated cancer rejection is a facet of autoimmunity, where the target tissue is the cancer itself. The induction of immune-mediated tumor tissue rejection represents an important conceptual approach to cancer immunotherapy and also remains an important goal in tumor immunology [4,5]. Antigens that function as tumor rejection antigens are considered self, nearly self or non-self [6]. The fact that a tumor antigen elicits a tumor-specific immune response does not necessarily mean that the immune response will cause the rejection of the tumor *in vivo*. The question remains as to which tumor antigen can or is better at inducing a clinically beneficial response [7]. Tumor-rejection antigen is therefore an operational term describing how well an immune response elicited against a tumor antigen will impact on tumor growth. Tumor antigens can be poor, intermediate or strong tumor rejection antigens, describing quantitatively the impact of the immune response on tumor growth [6].

**Figure 1:**
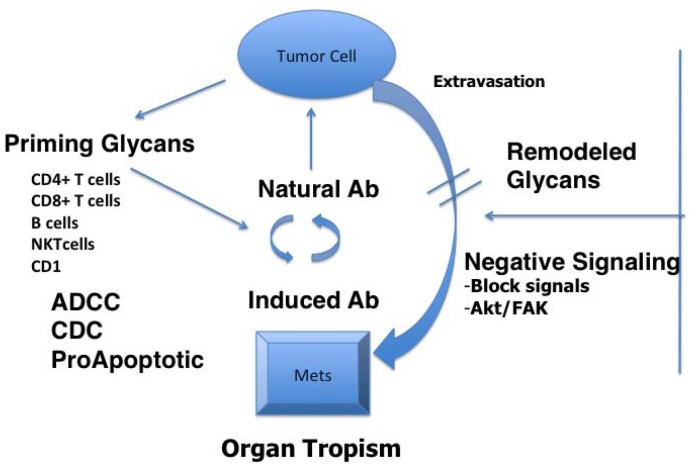
Immune surveillance targeting of tumor-associated carbohydrate antigens (TACA) allows both attack on tumor cells and interference with the tumor-generated immunosuppressive factors. Differentially expressed glycans mediate tumor cell dissemination and organ tropism. Carbohydrate antigens are bound by natural antibodies, thymus independent B-cell response generated antibodies and, rarely, by thymus-dependent responses. NK cells and CD1-dependent T-cells are also involved. A wide variety of antibody mediated effector mechanisms are at play—complement- and antibody-dependent cytotoxicity, direct proapoptotic effect, interrupting immunosuppressive signaling, migration, extravasation and organ tropism.

Among potential tumor rejection antigens are glycans expressed on glycoproteins and glycolipids. Aberrant glycosylation is a universal feature of cancer cells with some tumor-associated carbohydrate antigens (TACAs) considered tumor progression markers. A considerable body of evidence put TACAs amongst the most challenging of clinical targets for cancer immunotherapy [8,9], yet immune responses to glycans are noted that could lend to therapeutic strategies and approaches (Figure 1). TACA expression on cancer cells is associated with organ tropism underlying extravasation and metastases, because of glycan receptors on organ tissues [10] or their role in survival. A requisite for metastases is cell survival. Anoikis resistance or survival in the absence of attachment to extracellular matrix (ECM) is a prerequisite for the development of tumor metastases [11,12]. Anoikis resistance has evoked special attention in cancer research because circulating tumor cells in the blood stream are resistant to it. Signaling cascades are intimately interconnected with TACA expression and interaction with the microenvironment. TACAs can regulate the interaction between integrin and Focal Adhesion Kinase (FAK), for example, which, in turn, regulates cancer cell adhesion and invasion [13,14,15,16,17,18,19,20]. Many of the targeted TACAs are found on structures upstream of FAK that can modulate the signaling through FAK [14,17,19,20,21], whereby anti-TACA antibodies might reset anoikis of tumor cells. 

Glycans are considered as priming agents for T-cells and for B-cells working in concert [22,23,24]. Natural antibodies and induced antibodies can mediate tumor cell killing and tissue destruction by several mechanisms that include complement-dependent cytotoxicity (CDC) [25], antibody-dependent cellular cytotoxicity (ADCC) [26] and through signal transduction pathways, leading to anti-proliferative activity or apoptosis [27]. Antibodies to TACAs have other attributes, such as negating negative signals to immune cells by forming immune complexes with shed TACAs or by blocking attachment of tumor cells to microenvironment constituents. Remodeling the glycan surface of tumor cells either by bio-engineering approaches to facilitate antigen uptake to improve tumor cell immunogenicity [28,29,30] or through inhibitors that affect glycosylation in general may exacerbate the action of antibodies and NK cells reactive with glycan signatures.

Much of what we know about immune responses to carbohydrates stem from examining immune responses to microbes and developing pathogen-based vaccines. The success of carbohydrate-conjugate vaccines in anti-microbial strategies has fueled expectations for their success as cancer vaccines, because the pathophysiological process of infection and neoplasia are profoundly affected by similar or the same carbohydrate forms. Some worm glycan antigens, for example, share structural features with host-like glycans and TACAs, including Le(X) (Galbeta1-4[Fucalpha1-3]GlcNAc-), LDNF (GalNAcbeta1-4[Fucalpha1-3]GlcNAc-), LDN (GalNAcbeta1-4GlcNAc-) and Tn (GalNAcalpha1-*O*-Thr/Ser). Anti-glycan antibody responses are a prominent feature of the immune response, for example, in patients infected with schistosomiasis that display the LeX, LDNF and LDN glycans. It is well known that helminths have immunomodulatory effects on their hosts. They characteristically cause a skew toward T_H_2 immunity and stimulate Treg cells, while simultaneously inhibiting T_H_1 and T_H_17 responses. Carbohydrate antigens can directly stimulate NK cells, without previous antigen sensitization or MHC restriction, to initiate lysis and to produce IFN-γ. Costimulatory signals provided by NK cells, together with the effects of NK cell-derived IFN-γ on B-cell differentiation, isotype switching and immunoglobulin secretion, ultimately result in augmentation of the IgG humoral response against T-cell-independent antigens. In this mini-review, we place into context the selected roles of TACAs reactive immune surveillance. In particular, we focus on glycan-mediated phenomena associated with tissue rejection as a model to understand the rationale of controlling of tumor cell growth by some immune modalities that target TACAs. 

## 2. Glycans as Tumor Antigens

The rationale for targeting TACAs was elegantly discussed in terms of tissue distribution and therapeutic importance [31]. The transition in glycosylation patterns of cancer cells reflect a myriad of processes that correlate with poor prognosis of cancer, affecting cell signaling and communication, cell motility and adhesion, angiogenesis and organ tropism. Both simple glycan structures and more complex TACAs play a role in these processes. Glycan structures on the tumor cell surface result from the combined action of glycotransferases and glycosidases. The carbohydrate antigens that have been found to be tumor-associated (Table 1) include the mucin related Tn, sialyl Tn and Thomsen-Friedenreich (TF/T) antigens, the blood group Lewis-related Lewis(Y), Sialyl Lewis(X) (SLeX) and Sialyl Lewis(A) (SLeA), and Lewis(X) (also known as stage-specific embryonic antigen-1, SSEA-1), the glycosphingolipids, Globo H, and stage-specific embryonic antigen-3 (SSEA-3), the sialic acid containing glycosphingolipids, the gangliosides, GD2, GD3, GM2, fucosyl GM1 and Neu5GcGM3 and polysialic acid. SLeX and SLeA, in particular, are carbohydrate molecules that mediate the adhesion between tumor cells and the endothelium. Overexpression of SLeX and SLeA is combined with poor prognosis and malignant relapse [32]. The interaction of the antigen SLeX on tumor cells and E-selectin on endothelial cells was shown to mediate adhesion of tumor cells to endothelial cells [33], possibly facilitating tumor cell invasion in blood microvessels, extravasation and migration into tissue. Additionally, colorectal tumor cells expressing SLeX might prefer the liver to form clinically evident metastases, due to interaction with local E-selectin [34]. SLeX expression and lymphatic microvessel density in primary tumors might predict disease recurrence, suggesting that for some cancer, both lymphatic and hematogenous metastasis is mediated by SLeX interactions [35,36].

**Table 1:** Common carbohydrate antigens targets from tumor biopsy specimens.

Carbohydrate antigens	Structure
Polysialic acid (PSA)	α2,8-/α2,9 NeuAc
Tn	GalNAcSer/Thr
sialyl Tn	Neu5Acα2-6GalNAcaSer/Thr
T antigen	Galβ1-3GalNAcαSer/Thr
Globo-H	Fucα1-2Galβ1-3GalNAcβ1-3Galα1-4Galβ1-4Glc
LeY	Fucα1-2Galβ1-4(Fucα1-3)GlcNAc-Galβ1-3(Fucα1-4)GlcNAc
SLeX	Neu5Acα2-3Galβ1-4(Fucα1-3)GlcNAc
SLeA	Neu5Acα2-3Galβ1-3(Fucα1-4)GlcNAc

Many cell-cell contacts are mediated by cell-surface glycans that are redundant on membrane constituents that effect signaling pathways associated with anchorage independent growth and anoikis. Upregulation of the *N*-glycan branching enzyme β-1,6*-N-*acetylglucosaminyltransferase V reduces cell-cell interactions within a tumor, promoting cell detachment and invasion of tumor cells [37]. Also, the expression of *O-*glycans containing an *N-*acetylglucosamine branch connected to *N-*acetylgalactosamine (GlcNAcβ1-6GalNAc), which is designated the core 2 branch, is closely correlated with highly metastatic phenotypes of several tumor types [36,38,39]. Core 2 *O*-glycans expressed on the cell surface can reduce cell-cell interactions [40]. Mucin-type *O*-glycans containing Core2 branches have distinctly different functions from those *O*-glycans that contain Core1 structures. Core2 branched *O*-glycans can have terminal structures that function as ligands for carbohydrate binding proteins [41]. Sialylated Core2 branched *O*-glycans without additional modifications exhibit anti-adhesive properties, which might be related to anoikis resistance. These results demonstrate that certain mucin-type *O*-glycans can either facilitate or attenuate cell adhesion to ECM components and lectin proteins, depending on the core structures and the structures of the non-reducing termini. Several studies revealing the role of core 2 *O*-glycans in immune responses in general show that core 2 expressions is a biologically significant change [42]. Furthermore, the core 2 *O*-glycan is a key backbone structure in forming some selectin ligands. β-1,6*-N-*acetylglucosaminyltransferase (C2GnT), expressed in cancer cells, may play important roles in tumor progression through circulatory system or direct invasion [36], since some of these structures inhibit NK cell activation [43,44,45,46]. Thus, *O*-linked oligosaccharides, in particular those containing core 2 branches, play vital roles in immune responses and may play dual roles in certain situations [40,47]. 

Although the effectiveness of some of vaccines targeting TACA has been demonstrated in a number of experimental model systems and suggested in several clinical trials, the mechanism underlying their mode of action is uncertain. The distinction between glycans expressed on glycoproteins or glycolipids can translate to differences in how the immune system generates responses [25]. Previous studies showed that the targets for effective CDC were glycolipids (e.g., GM2, GD2, GD3, fucosyl GM1, Globo H or LeY), whereas those in which no lysis was observed were carbohydrate (e.g., TF, Tn, sTn) or peptide (e.g., MUC1) epitopes carried by mucin molecules [25]. It is observed that some TACAs responsible for CDC are expressed on glycoproteins and glycolipids, such as Globo H and LeY. MUC1, Tn, sTn and TF, in contrast, are not expressed on glycolipids, making the distinction clear. It may be that other antibody-mediated mechanisms, such as ADCC, opsonization of tumor cells by leukocytes, induction of apoptosis and blocking of tumor cell invasion or metastasis also differ, depending on the biochemical and biophysical nature of the targeted antigen. Monoclonal antibodies [26] and human serum antibodies from MUC1 immunized subjects [48] can mediate ADCC of human cancer cells. 

## 3. Innate Recognition of Glycans That Lends to Tumor Suppression

Since tumor tissue rejection is the goal of cancer immunotherapies, broad-spectrum tumor associated antigens, like TACAs, are plausible targets once the problem of their low immunogenicity is solved. The fact that multiple proteins and lipids on the cancer cell are modified with the same carbohydrate structure creates a powerful advantage for TACAs as cancer targets in immunotherapy strategies. Thus, targeting TACAs has the potential to broaden the spectrum of target pathways recognized by the immune response, thereby lowering the risk of developing escape variants, due to the loss of a given protein or carbohydrate antigen. There is an emerging awareness that immune surveillance mechanisms that include antibodies and effector cells are intimately related to TACA reactivity that provides a template for developing strategies for cancer immunotherapy, because of the display of glycans in the context of pattern recognition [49,50,51,52]. Glycans can be clustered representing danger signals to the immune system. Pattern recognition receptors (PRRs) are sentinels of innate immunity that instruct adaptive immunity mechanisms by which long-lived lymphocyte responses are targeted to appropriate antigens [53,54]. Innate immune cells have evolved to sense microbial pathogens through PRRs, coupling pathogen recognition to innate immunity through glycan-dependent mechanisms [55]. The same mechanisms might be operative for glycans expressed on cancer cells. Natural carbohydrate reactive antibodies have been described that mediate tumor cell apoptosis in addition to modulating complement associated cell killing [27,56,57]. Preclinical studies support the hypothesis that antibody-induced responses against TACAs might have their greatest impact in the adjuvant setting, as antibody responses inhibit tumor outgrowth in metastatic models [58]. Such observations suggest that sustained immunity against TACAs should be beneficial to prevent the recurrence of disease, much like the natural ways of immune surveillance. Therefore, maximizing sustained TACA-specific humoral immunity is considered an important goal in developing effective antibody-based immunotherapies against cancer. 

Like antibodies, NK cells are partners in immune surveillance. Three predominant superfamilies of NK cell receptors (NKR) have been identified that can either inhibit or activate NK cell function: (i) killer immunoglobulin (Ig)-like receptors (KIR) that bind to classical class I MHC molecules; (ii) C-type lectin receptors that bind to non-classical class I MHC molecules or “class I-like” molecules; and (iii) natural cytotoxicity receptors for which ligands are currently not well defined (except for NKp30 binding to B7-H6 and BAG6) [59]. Interestingly, it is possible that some of the natural cytotoxicity receptors may be binding to glycolipids [60]. NK cells can directly lyse virally infected cells and tumor cells without prior sensitization and provide immunoregulatory cytokines that shape the adaptive immune response. Cytolytic signals, triggered by inhibitory and activation receptors on the cancer cell surface, regulate NK cell-mediated cytotoxicity and the production of chemokines and inflammatory cytokines that mediate the immune response. The expression of some TACAs lend to the evasion of NK cell immunity [43,44], while others activate NK cells [61]. Therefore, aberrant glycosylation, while a target for immune surveillance, can regulate negative signaling of NK cells. 

### 3.1. The Case for TACA-Directed Antibody-Mediated Tissue Rejection

The eradication of xenografts has been suggested as a model to provide important insights about the role played by immunity in mediating tumor tissue rejection [5]. Tissue destruction occurs with resolution of pathogenic processes (cancer, infection) or tissue damage and organ failure (autoimmunity, allograft rejection) [5]. While xenograft rejection is highly mediated through innate immune mechanisms, in tumor immunology, the primary focus for tumor tissue-rejection is focused on effector cell-mediated tumor rejection and, particularly, the definition of cognate T-cell subsets that define signatures for tumor cell rejection. Nevertheless, underlying the various mechanisms associated with the biology of tissue damage or rejection emerges as a common pattern in tumor tissue-specific destruction relevant to TACA targeting [5]. These patterns were elegantly reviewed by Marincola and colleagues [5], which include the postulates that: (1) Tissue-specific destruction does not necessarily only occur after non-self-recognition, but can also occur against self-or quasi-self-antigens. In the context of tumor targets, TACA reactive antibodies are constantly produced, being inherent in the innate and adaptive immunity. (2) The requirements for the induction of a cognate immune response differ from those associated with the development of its effector phase. Natural circulating anti-TACA antibodies are present and are known to be apoptotic to tumor cells. Therefore, antibodies can function as both judge and jury. (3) Although the mechanisms prompting tissue-specific destruction differ among immune pathologies, the effector phase converges into a common activation of adaptive and innate cytotoxic mechanisms. In this context, glycan-reactive T-cells might work in unison with NK cells and antibodies to target tumor cells. Furthermore, (4) adaptive immunity triggers a tissue-specific reaction, but it is not always sufficient or necessary for tissue destruction. Carbohydrate-reactive antibodies bind to both normal tissue and cancer cells. The binding to normal tissues does not necessarily lend to normal tissue destruction, but may facilitate microenvironment interactions that lend to tumor tissue rejection. Indeed, immune-based therapies have the potential to modulate the tumor microenvironment by eliciting immune system cells that will initiate acute inflammation that leads to tissue destruction [62]. 

Antibodies can mediate tissue rejection that validates targeting TACAs. A model for glycans as tissue rejection antigens includes the response to the xeno-carbohydrate antigen Galα1-3Galβ1-4GlcNAc-R (alpha-Gal) epitope. The majority of alpha-Gal antigens are built upon the Gal1, 4GlcNAc (type 2) chain, but other inner-core saccharide chains also exist, especially on glycolipids [63,64,65]. Naturally occurring anti-Gal antibody is produced as the most abundant antibody (1% of immunoglobulins) throughout the life of all individuals [66]. Natural antibodies, such as anti-Gal or anti-blood groups A/B antibodies, mediate hyperacute graft rejection and, thus, represent a major hurdle in xenotransplantation [67] and blood transfusions, respectively. In the initial stage of the rejection, anti-Gal IgG binds to R-Gal epitopes expressed on the surface of xenograft cells, triggering antibody-dependent cell-mediated cytotoxicity by human blood monocytes and macrophages. The IgM isotype of anti-Gal is believed to be responsible for the complement activation that leads to complement-mediated lysis of the xenograft cells. 

While early studies suggested an increased risk of cancer and poor prognosis associated with ABO blood groups, such assertions have not been verified in breast cancer patients [68], but in ovarian cancer, the presence of the B antigen was positively associated with ovarian cancer incidence, whereas blood group A was not associated with risk [68]. One widely-occurring change observed in a large variety of human cancers is deletion of the A or B epitope on tumor cells, associated with accumulation of their precursor H (LeY, LeB), which causes enhanced malignancy [69]. The blood group reactive lectin *Griffonia simplicifolia* (GS-I), which recognizes alpha-galactosyl moieties is recognized as a surrogate marker to identify tumor expressed antigens reactive with anti-Gal antibodies [70], and GS-I lectin is of utility to interrogate terminal α-GalNAc/Gal expression on human tissues [71]. 

The antibody-mediated tissue rejection model supports a rationale for targeting TACAs as tumor-induced antibody responses resemble autoimmune responses [72]. Hyperacute rejection is a complement-mediated response in recipients with pre-existing antibodies to the donor (for example, ABO blood type antibodies). Tolerance to autologous ABO blood group antigens seems to depend in part on peripheral control of antibody autoreactivity. However, normal human serum does contain “hidden” natural antibodies reactive with autologous ABO blood group antigens [73]. These naturally occurring antibodies, especially the anti-Gal response, might also have other clinical consequences for immunotherapy [74] in the context of tolerance [75,76], cross-presentation of tumor antigens [77] and increased immunogenicity of cell-based and protein-based vaccines [66]. Consequently, further research is required to develop the translational and clinical applications.

### 3.2. The Case for Glycan-Directed T-Cell Mediated Tissue Rejection

As T-cell-dependent antigens, proteins have long been seen as the primary target of adaptive immune responses. In contrast, carbohydrates are characterized as T-cell-independent (either Type 1 or Type 2) antigens [78]; yet, early studies demonstrated that T-cells could recognize carbohydrate antigens [79]. Post-translationally modified T-cell epitopes constitute a small fraction of both MHC-I- and MHC-II-bound peptides, and a number of modifications are identified as natural MHC ligands *in vivo* [80]. Computer-based sequence analysis suggests that only a minimal portion of experimentally verified T-cell epitopes are potentially *N*- or *O*-glycosylated (2.26% and 1.22%, respectively) [81] and T cells are demonstrated to react with processed glycopeptides and glycolipids often representing TACA [82]. Some types of carbohydrates seem to be processed and presented to T-cells by MHC-II [83,84] while others associate with the MHC-I groove [85,86,87]. The demonstration that T-cells can recognize non-protein antigens has modified ideas on the breadth of antigens capable of interacting with T-cells [88]. The size of the carbohydrate chains, as well as *O*- *versus N*-glycosylation varies depending on tumor histotypes. However, recent studies suggest that *O*-glycosylation (GalNAc) presentation on a peptide backbone, while inducing CD4+ T-cells can impact negatively on CD8+ T-cell stimulation [85,86,87]. Structures of MHC Class II/peptide complexes suggest analogies with helical carbohydrate structures that could fit the MHC Class II antigen-binding groove [84]. In some cases, carbohydrate directly stimulates T-cells. Specific T-cell clones have been generated from mice immunized with a meningococcal group C (alpha-2→9-sialic acid) polysaccharide-tetanus toxoid conjugate [90]. These clones were MHC-independent, but still needed contact with antigen presenting cells for optimal activation [90]. 

Crystal structure analysis of TCR-glycopeptide interactions validate that TCR can recognize glycans presented on a peptide backbone [91,92]. Existing structures display the key interaction of the core of the peptide ligand, with the TCR CDR3 region shaping a “cavity” often accommodating aromatic amino acid residues. The latter are successfully mimicked in size and conformation by short glycans, like TF or the monomer, Tn. The ability of T-cells to recognize mono- and di-saccharides attached to peptides with Ser or Thr might indicate that T-cells might be degenerate in recognizing glycopeptides [51]. It should not be surprising that sometimes glycopeptides offer no significant benefit as targets for cytotoxic immune response. In some cases, CTL, generated upon immunization with glycopeptide, preferentially kills target cells treated with glycopeptide compared to those treated with the core peptide. In other cases, it does not matter [93], and in some cases, it has been suggested that other glycan receptors are involved in T-cell targeting [94]. This is particularly evident in the work of Madsen *et al.* [89] that clearly suggest that natural processing of GalNAc on MUC1 might not be a suitable for activating CTLs against MUC1. In general, this may or may not matter, because (a) some activated CTLs are cross-reactive with both the glycosylated and non-glycosylated forms of the same peptide and (b) glycopeptides are of low abundance on tumor target cells [93]. 

Polyclonal CTL have been observed to kill target cells expressing glycolipid [82]. It has been suggested that glycopeptide-specific-restricted CTL and unrestricted glycan-specific CTL belong to different T-cell populations with regard to TCR expression [95]. Such results demonstrate that hapten-specific unrestricted CTL responses can be generated with MHC Class I-binding carrier peptides. It is possible that CTLs activated with non-glycosylated peptides can cross-react with glycopeptides and carbohydrate themselves. Such peptides have been referred to as carbohydrate mimetic peptides (CMPs) or mimotopes. Sequences and structural properties of CMPs have been discussed previously [96,97,98,99]. CMPs are known to generate T-cells cross-reactive with carbohydrates [100] and to tumor cells [76,100,101,102,103]. The similarity of extended peptide structure and carbohydrates that can fit within Class I or Class II groves has also been noted [97]. In addition, select amino acid residues can spatially overlap glycans attached to peptides in the Class I grove [99]. T-lymphocytes from CMP-immunized animals were shown to be activated *in vitro* by SLeX, triggering IFN-gamma production in a MHC-dependent manner. Stimulation by peptide or carbohydrate resulted in loss of L-selectin on CD4+ T-cells, confirming a Th1 phenotype. An enhancement in CTL activity *in vitro* against SLeX-expressing Meth A cells using effector cells from Meth A-primed/peptide-boosted animals was observed. CTL activity was inhibited by both anti-MHC class I and anti-L-selectin antibodies. These results further support a role for L-selectin in tumor rejection, along with the engagement by the TCR for most likely processed tumor-associated glycopeptides, focusing on peptide mimetics as a means to induce carbohydrate reactive cellular responses. Immunization of mice with this CMP reduced tumor cell growth in a transplanted mammary tumor model mediated, to a large extent, by CD8+ T-cells [58], but without any damage to normal tissue after vaccination with the CMP [104]. These observations are very important in understanding the complexity of the antitumor response, especially in terms of abnormal glycan expression patterns and developing strategies in vaccine design.

### 3.3. The Case for NK Cell-Mediated Rejection

Cell-mediated cytotoxicity is a primary effector function of NK cells. It has been known for a long time that NK cells play a major role in tumor immune surveillance by serving as the first line of antitumor immune defense [105,106]. The multifaceted steps early in NK immune surveillance include an orchestrated activation and recruitment to the tissue sites where they, perform effector functions, which may be associated with tumor reactive antibodies. Receptor diversity is crucial in allowing NK cells to respond effectively, mediating their effects through direct cytolysis, release of cytokines and regulation of subsequent adaptive immune responses [107,108,109,110,111]. NK cell lysis is regulated by a balance of intracellular signals transmitted via stimulatory and inhibitory cell surface receptors after specific binding to their respective target cell ligands [112,113,114]. Activation of endogenous NK cells bears limited clinical benefit, as most cancer patients are treated with chemotherapy, and their immune system is compromised. Consequently focus has been directed in recent years to first understand NK suppression mechanisms and how better to exploit NK cell functionality.

Antibodies promote NK cell activation through antibody-dependent cell-mediated cytotoxicity. The best example of combining an anti-GD2 antibody with NK cells is in neuroblastoma (NB) [115]. Treatment of patients with high-risk NB with monoclonal antibodies targeting the disialoganglioside surface antigen GD2 has resulted in lower recurrence rates and improved overall survival [116,117,118,119]. In addition to complement-dependent cytotoxicity, the anti-GD2 monoclonal antibody 3F8 achieves NB killing through antibody-dependent cell-mediated cytotoxicity mediated by myeloid and NK cells [117]. To combine specific antibody-mediated recognition of NB cells with the potent cytotoxic activity of NK cells, clonal derivatives of the clinically applicable human NK cell line NK-92 that stably express a GD2-specific chimeric antigen receptor (CAR) comprising an anti-GD2 ch14.18 single chain Fv antibody fusion protein with CD3-ζ chain as a signaling moiety has been described [120]. CAR development in general is a hot topic area in immunotherapeutics, but mostly in developing T-cells for adoptive therapy [121]. The therapeutic efficacy of endogenous NK cells depends on the effectiveness of NK-activating agents to mobilize sufficient numbers of these cells to tumor sites [122]. The clinical utilization of NK cells is considered at the forefront of cancer therapy. It should be clear that adoptive transfer of NK cells should lead to high levels of circulating NK cells, but that does not necessarily translate into mediating tumor regression [123]. This may result from expression of glycans on the tumor cells in addition to glycans shed from the tumor cell surface. 

A variety of studies have linked the nature of signaling with the glycan ligand NK receptor paring. In this context, interest has focused on the *N*-glycan biosynthesis of glycoproteins and, in particular, branching enzymes, such as *N*-acetylglucosaminyltransferase III (GnT-III), GnT-IV, GnT-V and a1-6 fucosyl- transferase (a1-6FucT) [124,125], that can regulate the further processing of the *N*-glycan structures, which play a pivotal role in tumor development, metastasis and invasion. Heparan sulfate proteoglycans play a role in NK cell initial recognition and activation [126,127]. The interaction of SLeX antigen with lectin-like receptors on NK cells also triggers cytotoxicity [128,129]. Clustered glycoconjugates sharing the common structure motif trisaccharide Le(x) [130] can enhance cytotoxicity specifically by CD16+ NK cells. GlcNAc-terminated glycoclusters are found to be potent inhibitors of receptors on natural killer cells [131]. *N*-acetyl-d-glucosamine (GlcNAc) transferases, MGAT3 and MGAT5, have major involvement in linking terminating residues on glycans. MGAT5 is responsible for adding β1-6 GlcNAc residues and forming branched structures, which are especially abundant in cancer tissues with high metastatic potential. MGAT3 catalyzes the addition of β1-4 GlcNAc residues and forms a bisecting structure that disables further addition of GlcNAc by other glycosyltransferases, like MGAT5. Expression of terminal GlcNAc is perceived to inhibit NK function supported by experiments in which siRNA targeting these glycosyltransferases in tumor cells are observed to increase NK cell activity towards tumors [132]. 

Some transformed cells evade immune surveillance and become resistant to NK cell cytotoxicity, mainly because some shed TACA inhibits NK cell activation [45], leading to established primary tumors [133,134,135,136]. In renal cell carcinoma, the presence of higher gangliosides correlates with systematic metastasis. Disialosyl globopentaosylceramide (DSGb5) was identified previously as one of the major gangliosides from renal cell carcinoma (RCC). Siglec-7 (sialic acid-binding Ig-like lectin-7), expressed on NK cells as an inhibitory receptor, has a striking preference for internally branched α2,6-linked disialic gangliosides, such as DSGb5 [135]. These results suggest that DSGb5 expressed on RCC cells can downregulate NK cell cytotoxicity in a DSGb5-Siglec-7-dependent manner and that RCC cells with DSGb5 create a favorable circumstance for their own survival and metastases [135]. Consequently, despite the enthusiasm of using NK cells in adoptive transfer protocols, in most cases, NK functionality needs to be reset by remodeling the tumor glycan surface. The remodeling can lead to activation of endogenous NK cells with anti-tumoral function. Studies exploring such possibilities are warranted and under research.

### 3.4. Remodeling Glycan Signatures

Strategies for cell surface “glycoform remodeling” promise to facilitate the investigation of carbohydrate mediated cell-cell interactions [137] and as cancer vaccines [138]. Expression of the human α1,2-fucosyltransferase, for example, in transgenic pigs modifies the cell surface carbohydrate phenotype and confers resistance to human serum-mediated cytolysis [139]. The T-cell-independent process of delayed xenograft rejection is suggested as a model for glycan remodeling, which augments NK cell activity [140]. While natural antibodies against alpha-Gal epitope cause hyperacute rejection of pig organs in primates, evidence for the role of alpha-Gal in the NK cell-mediated xeno-response has been contradictory [141]. Therefore, while logic would dictate that glycan remodeling facilitates an improved immune response [138], the nature of these responses might be limited to antibodies and T-cells. 

Nevertheless it was argued early on that inhibition of *N*-linked oligosaccharide processing in malignant cells is associated with increased susceptibility to natural immunity [142]. Interference with *N*-glycosylation has been shown both to reduce the membrane expression of MHC class I and to increase the *in vitro* sensitivity of tumor cells to NK cell killing. It was long recognized that compounds that inhibit glycosylation pathways could affect the growth of tumor cells in tumor bearing animals. Castanospermine, swainsonine and tunicamycin block different steps in the pathways of glycoprotein processing that affect tumor cell dissemination and tumor colonization. This suggested blocking at one of at least two steps could have beneficial effects on tumor cell growth. The antimetastatic effect of tunicamycin may be related to interference in tumor cell-extracellular matrix interactions, whereas treatment with castanospermine or swainsonine appears to block at a stage distal to initial tumor cell arrest [143]. Swainsonine, in particular, is interesting, as it inhibits the formation of *N*-linked complex oligosaccharides with this inhibition correlative with enhancement with NK cell function. Consequently, inhibitors of *N*-, as well as *O*-linked glycosylation need to be expanded, because they should be useful for the treatment of cancer by effectively resetting NK functional activity by disruption of negative signals; given that inhibitors can be specifically targeted to tumor tissue [144]. More recently, it was shown that glycosylation regulates NK cell-mediated effector function through the PI3K pathway [132].

Antibodies might also regulate glycan expression patterns in an undefined way that enhances NK activity. The orchestration of glycan remodeling and galectin-1 upregulation by the tumor suppressor p16^INK4a^ in pancreatic carcinoma cells to reconstitute susceptibility to anoikis underscores the potential and tight control of this lectin [145]. Anti-glycan antibodies can function like lectins, mediating cell death signals [58] and cell growth signals [146]. Other galectins can promote NK cell-mediated anti-tumor activity by expanding unique phenotypes [147]. Co-culture of naive NK cells with macrophages from Gal-9-treated mice resulted in enhanced NK activity, although Gal-9 itself did not enhance the NK activity [147]. Antibodies can do the same. Clinical studies have indicated a role for anti-ganglioside IgM antibodies (including anti-GD2) in passive and active immunity against some cancers [148,149,150]. Their mechanism(s) of action is not clear, but a study in which mice transgenic for anti-GD2 IgM antibody were protected from EL4 metastasis and death indicated a role for IgM, complement and NK cells [151]. In these studies depletion of NK cells with anti-asialo GM1 rabbit serum reduced or abrogated the observed anti-tumor effects, suggesting that NK cells play a major role in tumor eradication or suppression [151]. It is possible that the GD2 model actually opens a window on a more general innate circuitry, which has just been further elucidated. Macrophages activated by Toll-like receptor (TLR) ligands appear to stimulate B cells, including through CD40-CD40L interactions, to a state of activation (CD69+ CD25hi, CD317+) in which they produce IFN alpha and stimulate NK cells nonspecifically [152]. 

## 4. Augmenting Responses to TACA

The clinical importance of targeting TACAs is highlighted by the success of carbohydrate-based vaccines against infectious diseases, by the role of TACAs in autoimmune phenomena and by the observed anti-TACA antibodies as clinical correlates of positive outcome seen in patients with cancer. Carbohydrate-based vaccines against *Haemophilus influenzae* Type b, *Neisseria meningitidis*, *Streptococcus pneumoniae* and *Salmonella enterica* serotype *Typhi* (*S. Typhi*) are already licensed, and many similar products are in various stages of development. Therefore, factors contributing to the successes and failures of these bacterial vaccines serve as guides to developing carbohydrate-targeting cancer vaccines. The practical benefits of inducing TACA-reactive antibodies in patients with cancer are further demonstrated by observations that patient survival significantly correlates with ganglioside-reactive IgM levels [149,153]. Low affinity natural IgM antibodies have been found indispensable for anti-viral responses [154,155]. An analogous role for natural antibodies as an innate anti-cancer surveillance mechanism has been suggested, but has been underappreciated, so far [156,157]. The fact that survival rates of cancer patients are correlated with low (intrinsic) affinity and low-titer TACA-reactive antibodies argues that more robust antibody responses may not be necessary.

The successful development of anti-microbial vaccines has proven that antibodies—particularly those targeting carbohydrate antigens—are ideally suited for eradicating pathogens from the blood stream and from early tissue invasion. Similarly, vaccines targeting TACAs may also prove beneficial in treating micrometastases. This may be the case since anti-TACA antibodies correlate with beneficial effects on the course of malignant disease and long-term patient survival [149]. However, *N*-acetylglucosamine branch in *O*-glycans (core 2 *O*-glycans) expressing cancer cells acquire highly metastatic phenotypes by surviving longer in host blood circulation [43,44,45]. The induction of TACA reactive antibodies and NK cells to leukemic cells might prove specially beneficial, since simple glycan profiles and commonly contained sialyl-T (NeuAcalpha2-3Galbeta1-3GalNAc) and disialyl-T (NeuAcalpha2-3Galbeta1-3(NeuAcalpha2-6)GalNAc) antigens as major O-glycans are observed on these cells [158] and receptors on NK cells bind to alpha2,3-NeuAc-containing glycoproteins [159]. Therefore, maximizing sustained antibody immunity against TACAs that express simple Core 1 and Core 2 (C2GNT-1) structures is an important goal in developing effective cancer vaccines to combat recurrent disease. 

### 4.1. Taking Advantage of Natural Antibodies

The ability of the immune system to identify and destroy nascent cancer cells and, thereby, function as a primary defense against cancer, is a long-standing debate. This raises expectations of therapeutic development of antibodies derived from the promise of multifaceted biological potency compared to stoichiometrically binding antibodies that just interfere with receptor binding. It is postulated that anti-carbohydrate antibodies are part of immune surveillance, just as they are a first line of defense against infectious agents. Natural antibodies may not only have a direct cytotoxic effect on intact tumor cells, but also bystander effects. In addition, they are known to contain low affinity self-reactive fraction representing a “grey area” of the tolerance to self. It has been proposed that this “grey” self-reactivity actually detects quantitative rather than qualitative changes of the antigenic landscape—a function especially suited to detecting unnaturally increased expression of TACA [160].

Antibodies that bind to a broad spectrum of TACA can reduce tumor cell dissemination by multiple mechanisms, including blocking the adhesion of metastatic cells to adhesion molecules and generally functioning as regulatory molecules to thwart signaling processes that underlie migration and autocrine and paracrine activities that grant immune privilege to cancer. FAK is a non-receptor tyrosine kinase that plays an important role in signal transduction pathways that are initiated at sites of integrin-mediated cell adhesions and by growth factor receptors. FAK is a key regulator of survival, proliferation, migration and invasion: processes that are all involved in the development and progression of cancer. FAK is also linked to oncogenes at both a biochemical and functional level. Moreover, overexpression and/or increased activity of FAK is common in a wide variety of human cancers, implicating a role for FAK in carcinogenesis. Given the important role of FAK in a large number of processes involved in tumorigenesis, metastasis and survival signaling, FAK should be regarded as a potential pathway target in the development of antibodies targeting TACAs that are associated with anoikis [21] and blocking adhesion. 

Determining populations of glycan reactive antibodies in the repertoire of natural autoantibodies could lead to developing immunotherapies targeting cancer without affecting normal tissues or resulting in adverse side-effects. Thus, the application of natural antibodies, like IVIg, has the potential to be a supportive therapy for the treatment of cancer metastases and provide an opportunity to probe yet undefined roles of natural antibodies relating broad-spectrum reactivity with anti-cancer functional properties. Most anti-glycan antibodies recognize epitopes of two or three sugars. Consequently, antibodies can cross-react with similar terminal structures. This property of recognizing epitopes “shared” by different molecules is characteristic of anti-glycan antibodies and can be considered an example of “antigen mimicry”. In this context, it would seem that anti-Gal antibodies should be reactive with the histo-blood group antigens, LeB and LeY. Blood group B individuals show reactivity to Tn antigen [161], and some anti-Gal antibodies are cross-reactive with the blood group B antigen [162]. Anti-Gal alpha(1,3)Gal antibodies are observed to react with mucin 1 (MUC1) found on the surface of human breast cancer cells [163]. Thus, natural occurring anti-Gal alpha (1,3)Gal antibodies found in all human serum can react with self (MUC1) peptides expressed in large amounts on the surface of tumor cells, but not on normal cells. These findings are of interest and serve to explain reported findings that human cells can, at times, express Gal alpha(1,3)Gal; such expression is suggested as an artifact in that anti-Gal alpha(1,3)Gal antibodies react with mucin peptides [163]. 

The cross-reactivity of anti-Gal antibodies has been exploited in cell therapy, where autologous cells processed to express alpha-Gal epitopes result in anti-Gal-mediated, *in vivo* targeting of autologous tumor vaccine to antigen presenting cells (APC) [77,164]. Transfection of cells with the enzyme 1,3galactosyltransferase (1,3GT) with concomitant expression of the Gal epitope followed by immune complex formation by anti-Gal antibodies should increase transport to lymph nodes and processing of anti-Gal complexed vaccines internalized by APC. Anticipated results include an effective activation of vaccine-specific CD4(+) and CD8(+) T-cells and high cellular and humoral immune response [77]. While manipulating the pre-existing anti-Gal response may facilitate an efficacious vaccine response through antigen spreading to antitumor T-cell response, truly tumor-specific antigens are needed to contribute decisively to tumor regression [165].

However, some antibodies display exquisite specificity, like those directed toward the TF antigen [166]. Postpartum, carbohydrate structures on the cell walls of the gastrointestinal flora evoke natural antibodies of presumed TF specificity. These antibodies may provide an early barrier against TF-carrying tumor cells. The widely used regimen of neoadjuvant chemotherapy is demonstrated to stimulate the immune response to TACA in some patients, as reviewed by Andre *et al.* [167]. Small retrospective studies have suggested that post-chemotherapy lymphocyte infiltrates could be associated with better outcome in patients who did not reach pathologic complete response [167]. The high levels of anti-TF antibody before surgery is another example in which antibody targeting is associated with a better survival of stage II breast cancer patients [168]. This may indicate that the selection of immunopotentiating regimens of neoadjuvant chemotherapy might be beneficial for the host in conjunction with the functional activity of natural anti-cancer antibodies. 

On the other hand, the detectable spontaneous immune responses to T and Tn antigens are not necessarily efficient, since the expression of these antigens correlates with worse prognosis, mostly because of increased metastasis. The reason may be an escape of some cancer cells from the control by immune responses to TACA, like T, Tn and sialyl-Tn. It is also possible that the correlation with higher grade and metastasis is due to the observation that some tumors are resistant to immunoediting. It would be interesting to differentiate between primarily TACA-negative tumors and secondarily negative tumors that arise due to immunoediting. It is likely that specific suppressive influence of the tumor on the production of TF antibodies is associated with the stage and grade of the tumor. Postoperatively, these antibodies rebound, as do lymphocyte counts [169]. The observation of positive correlation between the level of TF antibodies and the count of lymphocytes in TF-responders appears to reflect the adaptive immune response and provides a further explanation for the involvement of anti-TF IgG in cancer-associated immunosuppression. However, the possible protective mechanism of TF antibodies in cancer has yet remained unclear, as is the role antibodies play in the natural anti-cancer defense system. The signs of tumor-immune system interaction, together with the ambivalence of the results, draw attention to the hypothesis that immune surveillance may be just an epiphenomenon of the “knowledge of self” or, at least, still very early in the process of evolutionary optimization. The tools are there, but maybe they are yet to be tuned.

### 4.2. Bridging Humoral and Cellular Responses

Because of their characteristic immunogenicity and/or immunotolerance, most TACAs fail to induce T-cell-mediated immunity that is critical for cancer therapy. Approaches to overcome this limitation or improve their immunogenicity include coupling covalently TACA to proper carrier molecules to form clustered or multi-epitopic conjugate vaccines, coupling TACAs to a T-cell peptide epitope and/or an immunostimulant epitope to form fully synthetic multi-component glycoconjugate vaccines [138]. Polyvalent vaccines containing a variety of tumor-associated antigens are being tested under the hypothesis that a greater number of antigens in a vaccine will increase the probability of containing the correct antigen(s) to stimulate an effective anti-tumor response. The case for a polyvalent cancer vaccine to induce antibodies to TACAs has been made [170], although, in general, there may be more heterogeneity in antibody responses to polyvalent vaccines than that anticipated with monovalent vaccines [171]. More recent studies on carbohydrate-based vaccines are essentially modifications to the basic premise of conjugate formulations [172,173,174,175].

The recognition that T-cell receptors can interact with glycopeptides has facilitated concepts for new antigens being developed to activate anti-tumor responses. The feasibility of T-cell antigens design based on carbohydrate structures is strongly supported by crystallography of several HLA/peptide complexes. These include designer glycopeptides to facilitate CTL activation [176], glycan modification of antigens to target to APC to enhance both CD4+ and CD8+ T-cell responses [177,178,179]. One of the more important glycan decorated tumor antigens is human mucin 1 protein (MUC1). Attempts to develop MUC1-targeting cancer vaccines based on carrier-conjugated unglycosylated MUC1 tandem repeat peptides or carrier-conjugated glycosylated epitopes have been largely unsuccessful. Problems here partly relate to the conformational differences between non-glycosylated vaccine sequences and tumor-expressed, aberrantly glycosylated MUC1. Moreover, densely glycosylated MUC1 glycopeptide might be inefficiently processed by antigen-presenting cells, which ultimately means T-helper cells and CTLS aren’t highly activated. 

More promising results in tumor models have been reported using a two-component vaccine approach based on an MHC I glycopeptide and a T-helper epitope [180]. A multicomponent vaccine comprising a glycosylated MUC1-derived glycopeptide covalently linked to a T-helper epitope and TLR immunoadjuvant elicited potent humoral and cellular immune responses, effectively reversing tolerance and demonstrated potent anticancer effects. The vaccine candidate comprises the thiobenzyl ester of Pam3CysSK4 as a TLR2 ligand adjuvant, together with the composite T-helper epitope and aberrantly glycosylated MUC1 peptide, CKLFAVWKITYKDTGTSAPDT(αGalNAc)RPAP, formulated into phospholipid-based small unilamellar vesicles. To test its effects *in vivo*, the tripart vaccine was administered to experimental mice and the animals challenged with MUC1-expressing mammary tumor cells after 35 days. A week after the cancer challenge, the mice were given another vaccine boost. Control mice were administered with vaccine constructs comprising either the unglycosylated vaccine or subunits of the overall vaccine structure, *i.e*., just the glycopeptide or the adjuvant. Immunization with the multicomponent vaccine led to significant reductions in tumor burden and weight when compared with treatment using either empty liposomes or immunization with a control vaccine that didn’t contain the MUC1 glycopeptide epitope or an unglycosylated multicomponent candidate. Immunization with the primary tripartite candidate also elicited robust IgG antibody responses against the MUC1 glycopeptide, including a mixed Th1/Th2 response.

However, there are aspects of MUC1 that are largely ignored in the literature that might impact on its utility as an immunogen. Recently it was found that several of the tumor-related glycoforms of carcinoembryonic antigen, and MUC1 might affect CLR signaling and DC differentiation. These are specific ligands for the pattern recognition receptors DC-SIGN [181] and macrophage galactose-type C-type lectin (MGL) [182], expressed on DCs. MGL1/2-positive cells are interesting, as they represent a distinct sub-population of macrophages, having unique functions in the generation and maintenance of granulation tissue induced by antigenic stimuli [183]. MGL1 is postulated to be actively involved in inflammatory processes [184]. Consequently, Tn glycans on MUC1 that bind MGL might instruct DC to drive Th2-mediated responses, which, unlike those of Th1 effector cells, are thought not to contribute to tumor cell eradication. This has several ramifications. Cancer patients with MUC1 expression profiles may exhibit a Th2-skewed cytokine profile within blood and tumor-infiltrating lymphocytes. This Th1/Th2 imbalance would coincide with disease progression and immunotherapy response. Various lines of evidence suggest that *in vivo* skewing of T-cell responses toward a Th2 type is an important mechanism of immune evasion in cancer patients [185,186,187]. Terminal glycan structures shared by both host and parasitic helminths include LeX, LDN and LDNF and the truncated *O*-glycans known as the T (Galβ1-3GalNAc*α*1-*O*-Thr/Ser) and Tn antigens, all glycan antigens that may interact with host lectins that skew the immune response to Th2 profiles [188]. This skewing may limit the efficacy of immunotherapeutic approaches [189]. Immunization with formulations that reflect a Th2 bias of the native antigen might only exacerbate the Th2 response. Ensuring induction of a strong type 1 response may be critical to the development of effective cancer vaccines. 

MUC1-derived non-glycosylated peptides are also demonstrated to mimic carbohydrate antigens that include the Gal epitope [76]. Non-glycosylated peptides that mimic TACAs are noted to induce both humoral and cellular responses to tumors. CMPs can induce cellular responses, including CMP- and TACA-reactive Th1 CD4^+^ and tumor-specific CD8^+^ cells [100,101], and CMPs can prime for memory responses to TACAs [190]. We have demonstrated that a single CMP can bind to antibodies with differing TACA specificities that, upon immunization, can induce divergent antibody responses that recognize a range of TACAs [191]. Thus, this important and novel feature of CMPs effectively broadens the repertoire of reactive antibodies without inducing autoimmunity in animal models. The capacity to induce a carbohydrate-cross-reactive humoral, a Th and a CTL response with one single CMP is clearly a unique property of this approach. The observations that CMPs can induce both antibody and cellular responses in the absence of autoimmunity emphasize the feasibility of CMP-based vaccination strategies and the potential benefits of maximizing their effectiveness. Furthermore, CMPs can be encoded into DNA and viral vectors to enhance long-term immunity, which precludes the need for repeated TACA-based vaccination to maintain immune surveillance. This approach has led to a phase I study of a carbohydrate mimetic peptide (manuscript in preparation) in stage IV breast cancer subjects. This CMP shares homology with a region of MUC1, but involved reverse engineering using antibody and lectin templates as the basis for CMP development [104]. 

The mimicking of MUC1 non-glycosylated peptides with the Gal epitope might also have unintended consequences. For example, mimicry might lend to confusion in deciphering the difference in natural antibody levels to MUC1 and clinical outcomes to MUC1-based vaccines if anti-Gal antibodies cross-reactive with MUC1 are not considered [192]. In addition, this mimicry might also skew Th2 type responses to MUC1 vaccines, which is contradictory to the present paradigm that stresses Th1 responses to MUC1 and other tumor associated antigens. In fact, it is easy to see that as MUC1 expressing cancer cells emerge, the Th2 response becomes set. Vaccines that are MUC1-based might only stimulate B-cells and T-cells that are already primed as the Th2 type, exacerbating what might be akin to “original antigen sin” or an amnestic response to MUC1 of Th2 type [193]. In addition, while transgenic mice expressing human MUC1 are perceived to be of importance to understand the immune response to MUC1 in humans, it is often overlooked that these transgenics also express murine MUC1 in which T cells generated to human MUC1 peptides cross-react with naturally expressed murine MUC1 peptides. This cross-reactivity is seldom discussed and has the potential to confound results.

## 5. Conclusions

Glycans or TACAs are important targets for cancer immunotherapy, as suggested by immune surveillance mechanisms. TACAs display important biological effects in tumor biology and tumor immunology. Most importantly, the recognition properties of glycans by immune effector cells have suggested translational strategies in immune therapy. The diversity of regulatory mechanisms involving glycans expands the range of possible effects of TACA targeting immunotherapeutic approaches. Anti-TACA antibodies, thus, may be involved in more than direct tumor cytotoxicity. Although the exact mechanism may represent a cascade of steps that are still to be established, immunization targeting TACAs has already been shown to yield antitumor effects mediated by NK cells or through neutralization of tumor immunosuppressive factors in the form of soluble gangliosides. Future work should clarify the points of involvement of antibody/carbohydrate interactions in modulating tumor growth and facilitating innate surveillance mechanisms.

The abrogation of negative regulatory signals imposed by glycans and the maintenance of the activated phenotype of NK cells can significantly enhance NK cell activity against solid tumors. Manipulating the balance between inhibitory and activating NK receptor signals, the sensitivity of target cells to NK cell-mediated apoptosis and NK cell cross-talk with other immune effector cells might hold therapeutic promise [194,195]. Efforts to modulate NK cell trafficking into inflamed tissues and/or lymph nodes and to counteract NK cell suppressors, might prove fruitful in the clinic. However, a greater understanding of how to downregulate negative signaling, the benefits of combination therapy, characterization of the functional distinctions between NK cell subsets and the design of new tools to monitor NK cell activity are needed to strengthen our ability to harness the power of NK cells for therapeutic aims. 
